# Supplementary material for: Assessment of exposure to zoonoses and perceptions of zoonotic transmission surrounding the Bwindi impenetrable forest, Uganda
Source: PLoS Negl Trop Dis. 2025 Nov 17;19(11):e0013701. doi: 10.1371/journal.pntd.0013701 (PMC12633871; doi:10.1371/journal.pntd.0013701)
Supplement: S1 Text — (DOCX) [file pntd.0013701.s003.docx]

Focus Group Guide

*The consent script will be read aloud by the focus group moderator before each focus group session and will be provided in paper format to each participant. Sections in italics are instructions for the focus group moderator.*

**CONSENT TO PARTICIPATE IN FOCUS GROUP**

**Title of study:** EpiCenter for Emerging Infectious Disease Intelligence

**Investigator:** Dr. Birungi Mutahunga Rwamatware (Bwindi Community Hospital), Dr. Christine Johnson, University of California Davis (UC Davis)

**Focus Group:** Emerging Infectious Disease Exposure and Activities Related to Risk

## INTRODUCTION AND PURPOSE

You are being invited to join a research study. The purpose of this study is to better understand your community’s interactions with wild and domestic animals, and your knowledge of potential disease risks associated with these interactions. This research activity is understanding the origin of emerging infectious diseases and their prediction. Emerging diseases include diseases that are new (like COVID-19) or diseases that were uncommon but are now causing outbreaks. The diseases we study can be transmitted through contact with animals, insects that carry these diseases, or from humans with the same disease. We want to better understand how people get these diseases.

Before you decide if you want to participate, we will explain the research study to you. Once you understand what it involves, including the risks and benefits, you can make an informed decision. This is what we call informed consent. Your decision to take part in the study is entirely voluntary. This means that you are free to choose whether or not you want to participate as a research subject.

If you agree to participate in this research, you will be asked to take part in a group discussion, called a focus group, with 5-10 people from your community. The focus group will last about 60 to 90 minutes. You will be asked questions about animals within and nearby your community, activities that may bring you into close contact with these animals, and diseases these animals may help spread. We also would like to know where in your community you have contact with these animals and mosquitos. There are no right or wrong questions or answers in this discussion – what is important is for us to listen to your thoughts and opinions.

Audio of the interview will be recorded so we do not forget the important things that you say. We will only record your voice. After the session, these recordings will be written down.

Minors over the age of 12 may participate in the focus group if one parent is present during the session.

## PRIVACY AND CONFIDENTIALITY

To minimize the risks of breach of confidentiality, we will not collect your name or use your name or any identifying information in our recording transcripts. The digital recording of the group discussion will be transferred directly to a computer accessible only to study personnel and the recordings will be destroyed once we have transcribed all the information. To help protect the privacy of other members of your focus group, you should not share information about who was in your group or things they may have said during the discussion.

## BENEFITS

You will not benefit directly from your participation in this discussion, but by sharing your experiences through the focus group, you may help us to discover ways to reduce the risk of people catching these types of diseases.

## COMPENSATION

We will provide refreshments during the focus group session. At the end, you will receive a small token that is appropriate with local culture and customs such as a small grocery item (for example powdered milk, soap, or juice) not greater than 10,000 Uganda shillings as appreciation for your participation. We will not pay you to take part in this study or pay for expenses related to your participation, such as travel costs.

## RISKS

The risks of this research are minimal. Some of the questions might make you feel uncomfortable. You do not have to answer any of the questions you do not want to answer.

This research study will address topics that may be illegal or socially sensitive where you live. If you are concerned that discussing the topics addressed in this study may bring harm to you, your family, or your acquaintances, please consider these additional risks of participation when deciding whether to take part in this research study.

## RIGHT TO WITHDRAW FROM THE STUDY

***Participation in this research study is completely voluntary***. You are free to decline to take part in the project. You can decline to answer any questions and you can stop taking part in the focus group at any time. Whether or not you choose to participate, or answer any question, or stop participating in the project, there will be no penalty to you. We as the study scientists also have the right to remove you from the focus group at any time.

## IF YOU HAVE QUESTIONS OR CONCERNS

If you have any questions about this research, please ask them now. We will give you a copy of this consent form with a list of people you can contact for any future questions or concerns.

# If you have questions about this study, you may contact:

Mr. Nahabwe Haven: +256 772 544589

If you agree to take part in the research and allow the interview to be recorded, please verbally acknowledge that you would like to participate.

**SCREENING QUESTIONS**

1. *Ask any young participants their age to confirm they are over the age of 12. If they are a minor over 12, ask them who their parent is and confirm the parent verbally consents to the minor’s participation.*
2. Does anyone in this group have a close relative or employers/employees also in this group?
   - *Ask if we can have people come back for a different focus group to split up relatives from the same household (except minors and their parent) or employers from employees.*

**FOCUS GROUP GUIDE**

*Read aloud the consent form and obtain the consent before beginning.*

We have asked you to join us today to learn more about life in your community and the types of activities that may put those in your community, including you, at increased risk for being exposed to new diseases. We can’t talk to all of your community, but we hope that you can speak about both your experiences and your observations/knowledge of life in your community. Remember that you don’t have to answer all of the questions or participate in all of the discussions. We understand that you are experts about life in your community, and that is why we have come to you. We want to learn from you. There are no right or wrong answers – all of this has to do with different life experiences. Thank you for agreeing to join us today and let’s start with a small activity to get to know each other.

**Activity to get to know each other**

Introduction – Take turns introducing each person, including the following information:

- Identify each participant by number (1-10)
  - *Give each participant a card with their number. Prompt discussion from different people using cues such as "go ahead participant 6" or “did you having anything to add participant 3?”.*
- Age
- Occupation / Livelihood

*Opening exercise to "map" the community. The group works together to help the moderator draw a map of the important points of the community that relate to human/animal interactions. Start by drawing a few local landmarks: current location, the forest, the clinic, etc. Return to map to facilitate animal contact discussions in the next section.*

**Routine contact with animals and insects in the community**

We want to learn about the routine contact people in your community have with animals around them. So, let’s start with pets.

*As needed for the following questions, generate lists of animals by writing on a whiteboard, butcher paper, or similar so that the whole group can see the lists; alternatively post images/photos of animals to a board. Divide lists into pets, livestock, wildlife as appropriate to help guide conversation. Prompt discussion of additional animal species by showing photos and asking participants what they call that type of animal, and what they know about it.*

# What are the types of animals that people in your community keep as pets?

- - *Probes: Are there animals that you keep in your house that you do not eat? Keep asking if any others? Suggest common types of pets for the area.*

# What types of animals are kept as livestock around here? Any others?

- - *Probes: Ask about “exotic species” that we know about, such as: cane rats, civet cats*

# Where do people usually keep this type of animal? Even if you keep them in one type of place, think of all the variations you have seen in your community and tell us about them. Also please tell us which are the most common places these animals are kept. *Refer to the list of pet and livestock animals generated.*

1. **What other animals – that people do not keep as pets or livestock – might come into one’s house in your community? These might be pests and uninvited, but animals that sometimes enter people’s houses.**
   - *If bats/rodents do not get mentioned*: Are bats and rodents ever seen in homes around here? Can people distinguish different types of bats or rodents? Tell me more.
   - What are ways that people keep these uninvited animals or pests outside their homes? Anything else?
   - If these uninvited pests do get in, how do people get them out?
   - *Use animal photo ID cards as needed to get more info about species.*
2. **What other types of animals are seen in your community?** *Refer to the list of animals generated.*
   - *Probes: Use animal photo ID cards as needed to get more info about species or ask if certain types of animals are seen.*

# Do you or people in your community have problems with wild animals in your crop fields, harming your livestock, or eating the livestock’s food?

- - What are the most common types of problems you have?
  - What do you (or others in your community) do about it? Explain.
  - How are unwanted animals kept out of your crop fields?
  - Have you used HuGo to prevent or reduce crop raiding?

1. **Do you or people in this community touch or handle any wild animals? Which species?** *Refer to the list of wild animals generated.*
   - Which types and how many wild animals did people in your community touch last season?
   - *Probe:* Can you estimate how many? Less than 10, more than 50, etc.

# Are there places to buy wild animals in this area? Where are they and what species can be bought?

- - *Probe:* Price of wild animals purchased or sold?

# Do you or people in your community eat wild animals or use wild animals in ceremonies or for medicinal purposes?

- - What types of wild animals are eaten or used?
  - *Referring to the list of wild animals:*
    - Where do people get these animals? (i.e., Market, vendor, hunt yourself, find/collect). Alive or dead?
    - How much do these different wild animals cost? How is the price set?
  - How are these animals prepared for eating*? (Associate with list to figure out which are eaten.)*
  - How are these animals used for medicine? *(Figure out which are used for medicine.)*
    - *Probe:* What parts are used for what purposes? How are these parts prepared for use?
  - How are these animals used in ceremonies or for other purposes? *(Figure out which are used for other purposes.)*

# What kind of animals are hunted in your community? Please list as many as you can.

- - How has wildlife available for hunting increased or decreased over the past 5 years? How has your diet changed as a result?

# Can you tell us how these animals are hunted? What tools are used?

- - *Probe:* Are snares, traps available?

1. **What are the reasons for hunting each of these animals?** *Refer to the list of hunted animals.*
   - *Probe:* Income? To eat? To sell? Sport?
   - *Return to the list of animals that are hunted – these prompts might remind them of other animals that are hunted.*
   - Are there any wild animals that are hunted or captured here and sold or transported to other regions for sale? If so, explain which species and where they are transported.

# What other types of contact do people in your community have with animals?

*Probe about nuisance animals, or any other ways*

1. **Now let’s switch to mosquitoes. What types of mosquitoes do you see around here? Are there different types of mosquitoes? (***Provide picture cards for the mosquitoes and then ask participants to identify the pictures of the mosquitoes they usually see. Research assistants then can record the correct names of the mosquitoes based on the pictures provided).*
   - Do you or people in your community have mosquitoes in your houses?
   - Are there breeding sites within your community/near your household?

- *Probe: Broken pots, stagnant water, open pits/latrines where mosquitoes can breed*
  - When are the mosquitoes most bothersome?
  - What do you do to combat or protect yourself from mosquitoes?
    - *Probe:* Products? Strategies to repel insects? Use of mosquito nets? (and if no mosquito nets, main reasons why not)
  - What about mosquitoes in areas near your community – like the forest, or other areas of interest?
    - *Probe:* Are you bitten by mosquitoes when you travel to the forest? At what times are you bitten?

**Human interaction with forest/remote areas**

We are going to switch topics a bit, from your community and interaction with animals within your community, to how common it is for people to visit/enter the forest, and the types of activities that are done there.

Let’s start by thinking of the nearest forest to this community:

# How long does it take to get there? How do people get there?

1. **Think of your ten closest relatives/friends, including yourself: On a regular month, how often would you all visit the forest? (***Probe to find out if its on a daily, weekly, monthly or quarterly basis)* **Think of your friend who visits the forest most: how often does he/she go? How much time does she/he spend there? Where does she/he go?** *Mark locations on map.*

# What are the reasons for going into the forest?

1. **What animals so you see when in the forest?**
   - *Refer to lists and animal photo ID cards.*

# I want you to take some time to think about the forest near you. Think of what it was like 5 years ago – so if we are in [current year], think back to about [5 years earlier]. Can you tell me what changes you have observed in the forest since then? Explain.

- - How has it changed?
  - *Probe changes in landscape:* More trees cut or burned down? More people living in it or near it? Replanting?
  - *Probe changes in wildlife*: What animals do you see less of? What do you think the cause is? Do you see more of other animals? Which animals? Have animals moved from the forest into the village, or the reverse?
  - How have people’s interactions with the forest changed?

**Diseases in your community**

*Next, I want to talk about health and diseases in this community. Think about your family. Think about your friends in your community. Think about what you have heard happening in your community.*

1. **What diseases affect people in your community?** *Brainstorm and make a list.*
   - Have there been outbreaks of some of these diseases?
   - *Probe further on Malaria, Dengue, Zika, COVID-19, Ebola:* What do you know about these diseases and how they are spread?
   - *Probe:* What about unexplained hemorrhages, unexplained fevers with rash, babies born with smaller heads or physical deformities? Have you ever seen anyone with these symptoms?
   - Do you know of any diseases caused by mosquitoes? Can you tell me which they are?
   - Do certain seasons bring more illness? Have there been unexplained outbreaks of febrile illness in the last 5 years?
   - Do you know of any illnesses that humans get from animals in this region (wild or domestic animals)? If so, please describe.

# Have there been diseases affecting animals?

- - What about domestic animals? Wild animals? Do certain seasons bring more illness?
  - Have there been any unexplained outbreaks of disease in domestic animals in this area in the past few years? If so, please explain.
  - Have there been any unexplained outbreaks of disease in wild animals in this area in the past few years? If so, please explain.
  - Do you know of any illnesses that domestic animals get from wild animals in this region?
  - Do you know of any illnesses that animals get from humans/people in this region?

# Where do you and people in your community go when you are sick?

- - I assume there are many places – tell me about all of them, and how you choose where to go first, and how you decide where to go based on symptoms?
  - If someone has a serious disease, who is informed and where are they taken?

**Human Movement**

*Finally, I want to talk to you about movement within your community and where people may be traveling to.*

1. Where do you and people in your community travel to? What are the purposes of this travel?
   - *Probe:* Where do you travel for leisure? Where do you travel for supplies? How often? Where do you travel for business or other rare occasions?
   - *Together add to the map drawn earlier of where this community travels to.*
