# Supplementary material for: Assessment of exposure to zoonoses and perceptions of zoonotic transmission surrounding the Bwindi impenetrable forest, Uganda
Source: PLoS Negl Trop Dis. 2025 Nov 17;19(11):e0013701. doi: 10.1371/journal.pntd.0013701 (PMC12633871; doi:10.1371/journal.pntd.0013701)
Supplement: Attachment — Submitted filename: Comments_PNTD-D-24-01469.docx [file pntd.0013701.s004.docx]

**References:** There appear to be some errors in the references. For example, in lines 147-148 the authors refer to a free modulation translation approach and reference this with [30] – however, in the reference list, the reference that relates to this approach is actually number 29. This suggests there might be other errors if the numbering is out – could the authors please double check their reference list for errors?

**Lines 148-151:** Was the interview guide used as the basis for both the qualitative and the quantitative data? I have the impression that the open-ended questions were used for collecting qualitative data, and the closed-ended questions for quantitative data, but it would be useful to have this stated more explicitly if this was indeed the case.

**Lines 152-159:** It would be important to have some additional information on how the participants were selected and contacted and the sampling strategy more generally – how was the number of 153 determined?

**Line 180-181:** Were these transcriptions checked by any of the researchers or anyone involved in the data collection process? I would have concerns that misinterpretations could easily occur if the translator/transcriber was not present for any of the interviews and was not familiar with the interview context.

**Line 181:** Typo – I believe it should be “All transcribed text *was* analysed….”

**Lines 181-185:** How were the themes and subthemes created? Just a sentence or so to explain how the researchers interacted with the data, and whether the themes were prepared in advance of the interviews (e.g. themes already identified based on the topic of interest), or if they were created during the data analysis process. The sentence in line 184 seems to suggest that coding started after the key themes were identified, and I have the impression from the themes in the results that these were already prepared. Consider this in relation to my general comments on methodology.

**Lines 184:** I would prefer the wording “Data were coded using NVivo 12 Software according to key themes identified” – just to make it clear that it is the researchers who identified/created the themes, and not the software.

**Line 212:** I’m not entirely comfortable with a statement making this general a judgement of the participants on the basis of the data provided – to me this is a conclusion/interpretation made by the authors (not a result) and belongs in the discussion. It is reported that 33% of participants perceived that animals could transmit disease to humans – why is this considered a “low” level of awareness? What level would there have had to have been for awareness to not be considered low – 50%? Or 75%? It may be preferable to put this result into context, with something like “Given the close proximity of the participants to a human-animal interface, we would have expected a higher proportion of participants to be aware that disease can be spread between humans/animals” or something to this effect – but once again, this would be for the discussion, not the results.

**Line 215-217:** The numbers listed here do not match the numbers in Table 2. This is perhaps because the results given in the text are for the overall population while Table 2 is divided into rural and urban populations – but I still find this a bit confusing. The reference to Table 2 could be clarified or included in a different sentence.

**Line 218:** Could you please include in parentheses the more widely known names of some of these diseases (e.g. “jiggers”, “diarrheal”).

**Line 240-241:** Please review the syntax of this sentence – perhaps adding the word “with” between “illnesses” and “many” would help?

**Line 240-241:** Could you please include some supporting data for this statement? Either quantitative or qualitative.

**Line 245-247 (Table 2):**

- I would appreciate some explanation of what is meant by “overall zoonotic awareness” and how this was assessed. Is this based on one question alone or a composite of multiple questions? The overall awareness of participants is mentioned multiple times in the results and in the discussion so it is important to understand what is behind this.
- Aside from the first characteristic “overall zoonotic awareness”, the OR are not significant for any of the other variables. If they are included, it would be useful to have a sentence somewhere in the results noting that there was no significant difference between rural/urban populations regarding awareness of specific zoonotic transmission mechanisms or diseases, and perhaps speculating on the reason why this difference was observed overall but not for individual transmission mechanisms/diseases.

**Line 267:** For the word “respiratory”, do you mean “respiratory diseases” or is this a term used for a specific disease by participants? (in which case it would be helpful to include this in parentheses).

**Line 274:** Reword to avoid using the word “noted” twice.

**Line 275:** “Flue” should be “flu” or “influenza” (unless this is a specific term used by participants?). “Cough” is usually a symptom, not a disease, so appears out of place here (again, unless this is a translation of a term used to indicate a specific disease – in which case it would be helpful to know what it is).

**Line 131, 282:** Additional spaces between some words – please address.

**Line 291:** Please reword for grammar, this could be “… there was human contact with both wild….”

**Lines 291-321:** I would suggest the removal of the count of mentions for this theme. These seem to refer to the qualitative results – and I am not sure what added value they provide for this. Given the approach of the study and the qualitative nature of many of the responses, I don’t think frequency-based measures are helpful in indicating whether something is important or not – presumably for this kind of study, if even one participant mentions something, it is worth including. It is also not used in any of the other themes, so appears a bit out of place here.

**Line 293:** I am not quite sure that “marauding” is the right word here – is there another word that could describe what is meant?

**Line 296:** I’m not sure what is meant by “in most cases and in some instances” – perhaps consider rewording?

**Line 298-299:** Please check the syntax of this sentence – perhaps “disposal of remains and contact with animals can expose…” or “disposal of remains requires contact with animals that can expose…”

**Line 370-371:** This sentence (beginning “while”) needs completion.

**Line 392:** When words like “surprisingly” are used, I think it is important to say why this finding is surprising. How is the result different from what you expected and why did you expect that?

**Line 418:** Change “human to gorilla” to “humans to gorillas”
