# Supplementary material for: Assessment of exposure to zoonoses and perceptions of zoonotic transmission surrounding the Bwindi impenetrable forest, Uganda
Source: PLoS Negl Trop Dis. 2025 Nov 17;19(11):e0013701. doi: 10.1371/journal.pntd.0013701 (PMC12633871; doi:10.1371/journal.pntd.0013701)
Supplement: Attachment — Submitted filename: Review Bwindi impenetrable forest Uganda.docx [file pntd.0013701.s005.docx]

Title:

Study of anthropozoonoses and zooanthroponoses knowledge and perceptions of transmission risk pathways of rural and urban tribal communities surrounding the Bwindi Impenetrable Forest in Uganda.

Comments:

No mention of One Health practices or the importance of cross-sectoral and transdisciplinary approaches for zoonotic and zooanthroponoses prevention

Methods

Are the objectives of the study clearly articulated with a clear testable hypothesis stated?
-Is the study design appropriate to address the stated objectives?
-Is the population clearly described and appropriate for the hypothesis being tested?
-Is the sample size sufficient to ensure adequate power to address the hypothesis being tested?
-Were correct statistical analysis used to support conclusions?
-Are there concerns about ethical or regulatory requirements being met?

The study ethics was cleared through international university review board and the in-country review board and national Council of Science and Technology and participants were read an informed consent information and signed off.

The goal of the study was to better understand communities (rural and urban) contacts with wild and domestic animals and their knowledge of potential disease risks associated with these interactions. The reviewer recommends the authors state in the aim/goal that the study was conducted near the Bwindi Impenetrable Forest in Uganda a known hotspot for zoonotic pathogen spillover. The review suggests awareness be added to the study goal given table 2 of the results is about awareness of animal associated transmission risk factors and specific zoonotic diseases.

In the study (total number of participants = 153) qualitative methods of focus group discussions using a questionnaire interview guide were used. In the Rural focus groups, there were 90 people split into groups of 6-7 people across three rural districts of Kisoro, Kanungu and Rubanda. The group size for each focus group was appropriate but referring to table 1 there appears to be only two livestock farmers one from urban group and one from rural Kanungu. Table 1 should describe if agriculture/farming have livestock, what type of livestock or are hunters (what they hunt)? More description about participants contact with animals or ownership of animals is required. Also were any participants involved as guides for ecotourism associated with non-human primates etc? The reviewer did not have access to supplementary material Appendix 1 therefore could not review the interview guide.

The urban FGD included 63 people (live in Kabale City) about 50 miles away from the rural districts. There were 30 percent fewer urban participants in FGD than in rural groups. The two comparison groups urban or rural should have approximately the same number of participants given the study is assessing the odds (probability) of a zoonosis or zooanthroponosis knowledge and transmission pathways between different geographic locations (rural and urban) and exposure to animals/humans or animal products.

More information is needed about how the community members were recruited through local administration, community health workers and elders. The only inclusion criteria mentioned were being 18 years old or greater in age and residing in the community for at least five years to have adaptive knowledge. The CHW may have been biased in their selection choosing participants who had contacted a zoonotic disease. Was the sampling convenience or purposive sampling?

The main tribal group in the study were the Bakiga people therefore it is not possible to compare between Bafumbira and Bakiga people. It is noted that the Bantu people are the original forest dwellers and were not included in the focus groups. This tribe were hunter and gatherers and were evicted from the BINP in 1991 when the park was formed. The authors should discuss why one tribe mainly participated in the study and how they could have engaged other tribes in the study to have a better representation of the communities in this area. The socio-economic situation of the communities could also be described given those bordering Bwindi are the poorest in the region and have limited household income alternatives (Bitariho et al Conservation Science and Practice. 2022;4:e12761).

Was the interview guide (questionnaire) pretested with a few urban and rural people (representative of survey participants) to check the questions were clear and not ambiguous and culturally/gender appropriate? <https://www.fao.org/4/w3241e/w3241e05.htm>? Pretesting (piloting) of interview guides is recommended and best practice for studies.

The statistical analysis in the mixed methods descriptive study were appropriate though the confidence intervals for the odds ratios are required. Having an independent translator (not involved in data collection) transcribe the audio recordings to English reduced information bias that may occur if study FGD interviewers undertook this transcription. Additionally, the reviewer acknowledges study efforts to remove interpretation bias by having three researchers involved in the creation of themes and sub-themes related to perceptions and local understanding of the meaning of zoonosis.

Multivariable logistic regression was used to measure associations of risk factors with zoonotic understanding for variables that significantly improved the model fit.

Results

Does the analysis presented match the analysis plan?
-Are the results clearly and completely presented?
-Are the figures (Tables, Images) of sufficient quality for clarity?

The receiver recommends the inclusion of a table showing the main themes and sub-themes that were created from the focus group discussions transcribed text. The perceptions and local understanding of the meaning of zoonoses are provided as examples of the main themes. What was the local understanding of the meaning of zoonoses? Were the specific symptoms of a particular illness in humans associated with symptoms in animals (syndromic framework) or was it associated with case definition and test result? Were photos or drawings used by the interviewer to convey questions?

The figure 2 of community knowledge about transmission of specific zoonotic diseases using pictures to illustrate the animals, vectors and animal products is well presented. In line 240 the reviewer notes a correction is needed to state that “many participants being **unaware** that they transmitted malaria”.

The table 2 and table 3 need the Odds Ratio 95% confidence intervals to be included as it is important to determine if the OR confidence interval includes the null value OR =1.

Table 2 is described as “factors associated with awareness of animal-to-human pathogen transmission” . The reviewer suggests changing the description to awareness of risk factors for zoonotic transmission and specific diseases. How is overall zoonotic awareness defined and calculated? Given the demographics of the study participants with 68.2 percent of participants in urban Kabale not having an occupation with animal contact, it is not surprising that the rural FGD participants were 3.53 times more likely to be aware that animals can transmit diseases to humans compared to urban participants.

The mode of transmission comment from FGD men, Ruhija (line 221-222) relating to eating food which a pig had also eaten and then suffering from diarrhea would need further investigation and could possibility be associated with transmission pathway for cysticercosis given *Taenia solium* is prevalent in pigs in many regions of Uganda?

It is interesting to note that bird flu (avian influenza) was mentioned once line 275 (zooanthroponoses) though respiratory diseases were included in table 2 therefore flu could be part of this specific zoonotic disease transmitted category. Another priority zoonosis is anthrax which was only mentioned in the discussion (line 395-396) of the paper associated with transmission from consumption of beef from sick cows. The One Health zoonotic disease prioritization for multisectoral engagement in Uganda (2017) included anthrax, zoonotic influenza viruses, viral haemorrhagic fevers, brucellosis, trypanosomiasis, plague and rabies. The Uganda One Health strategic plan 2018 -2022 had a goal to reduce the burden of prioritised zoonoses and AMR by 50 percent. The authors should discuss how their study may support Uganda to reduce the burden of prioritized zoonoses especially in this hotspot area.

In table 3 the demographic factors of rural, age and healthcare worker, were associated with overall zoonotic disease awareness. This table needs to define the “age” factor. From the OR = 0.40 it could be concluded that older participants are less aware of zoonotic diseases but given the small sample size it is important to include the confidence interval for this odds ratio result. It is logical to make the assumption that health care workers would have a significant greater zoonotic disease awareness given their training than other occupations (agriculture, livestock, farmers). It would be useful to further examine the ten health care workers education background and training.

The results description in lines 264-266 about proportion of participants with awareness of anthroponotic (animal- to- human) transmission mostly residing in rural setting (n=21/26) should be moved to the previous paragraph and it would be useful to know the occupations of these participants.

The transmission knowledge of diseases from humans to animals were skewed toward transmission to gorillas (n=12/26). It is noted that one of the study team is Dr Benard Ssebide from the Gorilla Doctors and had been involved with the wildlife authority. It would be useful to review the interview guide to better understand the questions associated with zooanthroponosis knowledge and conduct further studies to evaluate the Uganda Wildlife Authority training for Park personnel and general public (noted in last paragraph of discussion). Did all 12 participants who knew about this risk transmission pathway from humans to gorilla’s undertake this training in the past and were most in the FGD men from Ruhija?

The study results about contact with animals is well presented and an important finding in this study. The sentence in line 298-299 requires changing to read more clearly and could be “There is recognition that disposal of animal remains requires contact with animals that can expose people to diseases”. It is important to note that although some participants knew the animals could be infected with pathogens, they did not carry out appropriate infection prevention and biosecurity practices while handling carcasses. It would be useful to know if higher risk of zoonotic contact with animals was associated with gender? Is it mainly women who unset the animal traps? Gender was not found to be a demographic risk factor with overall zoonotic disease awareness as not included in table 3 results.

The study results on contact with meat from sick and dead animals are important findings. The statement that “people eat the meat because of poverty” by village health teams of southern division demonstrates the need to understand the drivers of behaviour and addressing the root causes of risk taking. A One Health approach is needed to support community health through improving livestock and wildlife health. Training of Community animal health workers (CAHW’s) is needed and the World Organisation for Animal Health WOAH (formally OIE) has recently launched competency and curriculum guidelines for community animal health workers which should be referenced. <https://www.woah.org/app/uploads/2024/09/woah-competency-and-curriculum-guidelines-for-cahws-071024.pdf>. Additionally, the WOAH and African Union commenced a project called ZOOSURSY to further build One Health collaborations for disease surveillance of priority emerging and re-emerging zoonotic diseases with implementation of the WOAH Wildlife health framework. The reviewer recommends that outcomes from this small qualitative study may support further research in this zoonosis hotspot area and more widely.

In the reporting of disease outbreaks results the willingness of participants to report diseases in humans and animals was found to be low (n=28/153, 18%) with half of those reporting contacting the community health workers. As there was no mention of community animal health workers or veterinarians for reporting disease occurrence in animals – perhaps participants were more focused on disease outbreaks in humans?

Conclusion

Are the conclusions supported by the data presented?
-Are the limitations of analysis clearly described?
-Do the authors discuss how these data can be helpful to advance our understanding of the topic under study?
-Is public health relevance addressed?

Overall comments

In the discussion several statements are missing references to support them – for example line 370 “our research augments previous studies into communities’ views of zoonotic diseases”.

The authors state that misconceptions about the origins of zoonotic diseases and transmission can be overcome through providing education citing a cross-sectional study in DRC following the end of the 2018 Ebola outbreak. The conclusion in this study recommended co-developed culturally sensitive and inclusive community evidence-based programs which compliments early anthropological insights from the West African Ebola epidemic about engaging communities and building trust. (Wilkinson A, Parker M, Martineau F, Leach M. 2017 Engaging ‘communities’: anthropological insights from the West African Ebola epidemic. Phil. Trans. R. Soc. B 372: 20160305. <http://dx.doi.org/10.1098/rstb.2016.0305>).

Increasing community knowledge and awareness alone is unlikely to be sufficient to successfully change human behaviours that drive zoonotic disease risk as described in a scoping review by Anna Durrance-Bagale et al (Drivers of zoonotic disease risk in the Indian subcontinent: A scoping review. One Health 2021. 13:100310). Co-designing context specific interventions using interdisciplinary and participatory methods are needed using One Health approaches. The cited (#31) Mangesho et al 2017 study among pastoralists in northern and eastern Tanzania also supports context specific interventions using One Health approaches taking into consideration social, cultural and economic aspects of communities.

A One Health approach with multi-sectoral and transdisciplinary collaborations, to zoonotic and reverse zoonosis prevention is urgently needed in this area as highlighted in this small semi-structured questionnaire FGD study. Currently strengthening of multisectoral collaboration for Mpox and anthrax prevention is occurring in the Kanungu district due to the location near DRC and cross-border interaction between school children. Training of approximately 200 school managers with IEC materials (posters and flyers) and a collaborative response action plan is centred on child-to-child strategy of empowering learners to share health message with peers, families and communities.

Further validation studies are required to evaluate the reason why some participants had awareness of reverse zoonosis transmission of disease from humans to gorillas. It is not evidence based to state that this reverse zoonosis awareness was due to targeted educational gorilla health programs surrounding BINP. A scoping review of all current educational programs and training relating to zoonosis and reverse zoonosis prevention and One Health practices is needed.

The recommendations need to be re-written in light of the reviewer’s comments. A One Health approach for co-developed culturally sensitive and inclusive community evidence-based programs for health system strengthening across human, animal/wildlife and ecosystem is needed. Additionally continuing to invest in long-term community livelihood projects that support poverty alleviation and improving the health of livestock and wildlife is recommended.
