# Supplementary material for: Assessment of exposure to zoonoses and perceptions of zoonotic transmission surrounding the Bwindi impenetrable forest, Uganda
Source: PLoS Negl Trop Dis. 2025 Nov 17;19(11):e0013701. doi: 10.1371/journal.pntd.0013701 (PMC12633871; doi:10.1371/journal.pntd.0013701)
Supplement: Attachment — Submitted filename: PNTD-D-24-01469 Response to Reviewer.docx [file pntd.0013701.s006.docx]

**Nahawe Haven**

**Bwindi Community Hospital,**

**P.O BOX 58, Kanungu, Uganda**

[hahotice@googlemail.com](mailto:hahotice@googlemail.com)

Dear PLOS Neglected Tropical Diseases Editorial Team,

**Re: Manuscript ID PNTD-D-24-01469**
**Title: “Assessment of exposure to zoonoses and perceptions of zoonotic transmission surrounding the Bwindi Impenetrable Forest, Uganda”**

Thank you for the opportunity to revise our manuscript. We appreciate the constructive comments and suggestions provided by you and the reviewers, which have helped us to improve the clarity and quality of our work. We have carefully considered each point raised and revised the manuscript accordingly.

**JOURNAL REQUIREMENTS**

**Comment:** Please ensure that the CRediT author contributions listed for every co-author are completed accurately and in full. At this stage, the following Authors/Authors require contributions: Scott Kellermann, and Tierra S. Evans. Please ensure that the full contributions of each author are acknowledged in the

**Response:** Contributions added

**Comment:** Tables should not be uploaded as individual files. Please remove these files and include the Tables in your manuscript file as editable, cell-based objects.

**Response:** This has been done as requested.

**Comment:** Figure 2; Please confirm whether you drew the images / clip-art within the figure panels by hand. If you did not draw the images, please provide a link to the source of the images or icons and their license / terms of use; or written permission from the copyright holder to publish the images or icons under our CC BY 4.0 license.

**Response:** Fig.2 was drawn by Eunah Preston, UC Davis, One Health Institute and used with her permission (email provided separately). This figure is published under the CC BY 4.0 license.

**Comment:** Figure 1; Please provide a direct link to the base layer of the map (i.e., the country or region border shape) and ensure this is also included in the figure legend; and provide a link to the terms of use / license information for the base layer image or shapefile. We cannot publish proprietary or copyrighted maps (e.g. Google Maps, Mapquest) and the terms of use for your map base layer must be compatible with our CC BY 4.0 license.

This Figure was drawn by a co-author and has been published under creative commons. This map was created using QGIS software. In the initial set up, we used Google Satellite as the base layer of this map as it is the best at showing the density of the Bwindi Impenetrable Forest National Park. Since the reviewer comments from PLOS she has been working to find a different, open-source base layer map, but so far has been unsuccessful in finding one that demonstrates the appropriate vegetation density that Google Satellite does. The data points plotted on the map were first collected as GPS coordinates at the time of each focus group discussion, imported to google earth, then exported as a KML file and added as a vector layer into QGIS. The base layer of the map was Google Satellite used through the QGIS software. We researched Google's terms and conditions of use for Google Earth images and found that their terms of use seem to indicate that it's ok to use Google Earth images and KML files as layers for maps in print as long as proper attribution is given.

We read through those guidelines here:

<https://www.google.com/help/terms_maps/>

<https://about.google/brand-resource-center/products-and-services/geo-guidelines/>

**Comment:** We note that your Data Availability Statement is currently as follows: "Data has been made available". Please confirm at this time whether or not your submission contains all raw data required to replicate the results of your study. Authors must share the “minimal data set” for their submission. PLOS defines the minimal data set to consist of the data required to replicate all study findings reported in the article, as well as related metadata and methods (<https://journals.plos.org/plosone/s/data-availability#loc-minimal-data-set-definition>).
**Response:** We have provided the data used to generate tables.

**Comment:** Please amend your detailed Financial Disclosure statement. This is published with the article. It must therefore be completed in full sentences and contain the exact wording you wish to be published.
- State the initials, alongside each funding source, of each author to receive each grant. For example: "This work was supported by the National Institutes of Health (####### to AM; ###### to CJ) and the National Science Foundation (###### to AM)."
- State what role the funders took in the study. If the funders had no role in your study, please state: "The funders had no role in study design, data collection and analysis, decision to publish, or preparation of the manuscript.".
**Response:** Amendment made

**Reviewers' Comments:**

**METHODS**
**Reviewer #1:**

**Comment:** This is a mixed-methods study - but at least for the qualitative aspects, the authors could consider referring to the Standards for Reporting Qualitative Research (SRQR), available at <http://www.equator-network.org/reporting-guidelines/srqr/>. There are some aspects of the methodology which to me are unclear and would benefit from more structure. For example:
- Rather than a section titled “Focus group discussion”, I would expect rather to have sections titled “Data collection” where the method of data collection is provided, “Participants”, where information on the choice of participants and sampling strategy is provided, and “Researcher characteristics”, describing the characteristics of the researchers (their experience in qualitative research, language, prior relationship with participants (if at all), positioning in relation to participants (e.g. outsider or insider) – as this information has implications for the interpretation of the results. Please see the SRQR for further details.

**Response:** This has been updated accordingly, and the suggested sub-headings have been used.

**Comment:** The abstract mentions a three-stage coding framework and the use of grounded theory – but there is no mention of this at all in the methodology section. In fact, aside from the abstract, there is no indication at all of what methodological approach was used to analyse the qualitative data. If grounded theory was used, this appears to me a bit counterintuitive, as the themes appear to have been defined almost in advance and in relation to the research topic of interest – this is a perfectly valid way of doing it, but does not really seem compatible with a grounded theory approach (which would involve the researchers’ identifying or generating the themes from the data itself). Some additional explanation and clarity on this would be appreciated. I would recommend including a paragraph on “Qualitative approach” where this could be described.

**Response:** This has been updated accordingly. A section that explains how qualitative data analysis was approached has been added.

**Reviewer #2:**

**Comment:** The study ethics was cleared through international university review board and the in-country review board and national Council of Science and Technology and participants were read an informed consent information and signed off. There was no mention of participants withdrawing from the study.

**Response:** This has been updated with a statement that no participants chose to withdraw.

**Comment:** The goal of the study was to better understand communities (rural and urban) contacts with wild and domestic animals and their knowledge of potential disease risks associated with these interactions. The reviewer recommends the authors state in the aim/goal that the study was conducted near the Bwindi Impenetrable Forest in Uganda a known hotspot for zoonotic pathogen spillover. The review suggests awareness be added to the study goal given table 2 of the results is about awareness of animal associated transmission risk factors and specific zoonotic diseases.

**Response:** This has been updated accordingly.

**Comment:** In the study (total number of participants = 153) qualitative methods of focus group discussions using a questionnaire interview guide were used. In the Rural focus groups, there were 90 people split into groups of 6-7 people across three rural districts of Kisoro, Kanungu and Rubanda. The group size for each focus group was appropriate but referring to table 1 there appears to be only two livestock farmers one from urban group and one from rural Kanungu. Table 1 should describe if agriculture/farming have livestock, what type of livestock or are hunters (what they hunt)? More description about participants contact with animals or ownership of animals is required. Also were any participants involved as guides for ecotourism associated with non-human primates etc? The reviewer did not have access to supplementary material Appendix 1 therefore could not review the interview guide.

**Response:** We have added a description of types of farming conducted in the region. The types of livestock are described in the qualitative results portion of the manuscript under “Contact with meat from sick or dead animals” (goats, cows, swine, and poultry). Similar contact with other animals was also described elsewhere in the qualitative sections including contact with rodents/cane rats, bats, and multiple types of non-human primates (gorillas, baboons, monkeys, and chimpanzees). We felt these descriptions provided better descriptions of the animal contacts than a footnote in the table and so left them for these subsequent sections of the manuscript. Description of hunted animals was not an explicit focus of this manuscript but is an interesting topic for future research.

**Comment:** The urban FGD included 63 people (live in Kabale City) about 50 miles away from the rural districts. There were 30 percent fewer urban participants in FGD than in rural groups. The two comparison groups urban or rural should have approximately the same number of participants given the study is assessing the odds (probability) of a zoonosis or zooanthroponosis knowledge and transmission pathways between different geographic locations (rural and urban) and exposure to animals/humans or animal products. More information is needed about how the community members were recruited through local administration, community health workers and elders. The only inclusion criteria mentioned were being 18 years old or greater in age and residing in the community for at least five years to have adaptive knowledge. The CHW may have been biased in their selection choosing participants who had contacted a zoonotic disease. Was the sampling convenience or purposive sampling?

**Response:** Focus group size and participant number was dependent on a combination of recruitment strategy and data saturation so was unlikely to yield exact balance between the groups. Differences in CHW networks, participant availability, and other factors contributed to imbalance between the numbers in each group despite intention for general equality between the groups. Sampling strategy description was updated accordingly in the manuscript.

**Comment:** The main tribal group in the study were the Bakiga people therefore it is not possible to compare between Bafumbira and Bakiga people. It is noted that the Bantu people are the original forest dwellers and were not included in the focus groups. This tribe were hunter and gatherers and were evicted from the BINP in 1991 when the park was formed. The authors should discuss why one tribe mainly participated in the study and how they could have engaged other tribes in the study to have a better representation of the communities in this area. The socio-economic situation of the communities could also be described given those bordering Bwindi are the poorest in the region and have limited household income alternatives (Bitariho et al Conservation Science and Practice. 2022;4:e12761).

**Response:** The Bafumbira and Bakiga are subgroups of the Bantu. Additionally, while not made explicit, the focus groups languages are inclusive of multiple local ethnic groups, particularly the Batwa, who were the primary group evicted from BINP ({Brubacher, 2024 #1910}). While conducted in two languages, the study team felt these languages allowed adequate participation from the diverse group of local participants. Potential reasons that Batwa did not chose to participate in the FGD’s may have been concern around taboos associated with wildlife hunting. Batwa had equal opportunity to participate in the study if they had wanted to. A sentence describing this was added to the manuscript.

**Comment:** Was the interview guide (questionnaire) pretested with a few urban and rural people (representative of survey participants) to check the questions were clear and not ambiguous and culturally/gender appropriate? <https://www.fao.org/4/w3241e/w3241e05.htm>? Pretesting (piloting) of interview guides is recommended and best practice for studies.

**Response:** We indeed conducted pretesting for the questionnaire to ensure that questions were asked the right way or achieved the intended research goals. This was done for the first three Focus Group Discussions. Questions were revised to cater for the needed changes and then used to collect data for subsequent interviews.

**Comment:** The statistical analysis in the mixed methods descriptive study were appropriate though the confidence intervals for the odds ratios are required.

**Response:** Confidence intervals have been added.

**Reviewer #3:**

**Comment:** The methods overall are well-described and appropriate. A few minor comments:
- Lines 128-129: please state if informed consents of illiterate participants were witnessed by an independent witness. If not, please confirm that the consenting procedure was approved as such by the local and national Ugandan ethics committees.

**Response:** The consent procedure was approved via the local ethics committee at Mbarara University of Science and Technology Institutional Review Board, the national ethics committee at Uganda National Council of Science and Technology, and UC Davis Institutional Review Board. Consent was accomplished through reading the full consent form and then participants who agreed to participate either signed or placed their thumb print. Attestation of witness of consent was not a required element of this consent.

**Comment:** Lines 152-155: please provide more information regarding how participants were recruited and selected for the focus groups. Was there an attempt to ensure the groups were representative of the local population (e.g., in terms of age, socioeconomic status, education, etc.)? Were residents selected at random to participate?

**Response:** A description was added about recruitment procedures to the manuscript.

**Comment:** Line 155: please describe or list the inclusion/exclusion criteria

**Response:** Updated accordingly.

**RESULTS**
**Reviewer #2:**

**Comment:** The receiver recommends the inclusion of a table showing the main themes and sub-themes that were created from the focus group discussions transcribed text. The perceptions and local understanding of the meaning of zoonoses are provided as examples of the main themes. What was the local understanding of the meaning of zoonoses? Were the specific symptoms of a particular illness in humans associated with symptoms in animals (syndromic framework) or was it associated with case definition and test result? Were photos or drawings used by the interviewer to convey questions?

**Response:** Summary table of themes and subthemes added.

**Comment:** This research focused more on elucidating general beliefs around human-animal disease spread so relationships between specific diseases and their syndromes were not comprehensively explored. Photos and drawings were not used to convey questions.The figure 2 of community knowledge about transmission of specific zoonotic diseases using pictures to illustrate the animals, vectors and animal products is well presented. In line 240 the reviewer notes a correction is needed to state that “many participants being unaware that they transmitted malaria”.

**Response:** Revised to clarify meaning

**Comment:** The table 2 and table 3 need the Odds Ratio 95% confidence intervals to be included as it is important to determine if the OR confidence interval includes the null value OR =1.

**Response:** CI’s added.

**Comment:** Table 2 is described as “factors associated with awareness of animal-to-human pathogen transmission” . The reviewer suggests changing the description to awareness of risk factors for zoonotic transmission and specific diseases. How is overall zoonotic awareness defined and calculated? Given the demographics of the study participants with 68.2 percent of participants in urban Kabale not having an occupation with animal contact, it is not surprising that the rural FGD participants were 3.53 times more likely to be aware that animals can transmit diseases to humans compared to urban participants.

**Response:** Revised.

**Comment:** The mode of transmission comment from FGD men, Ruhija (line 221-222) relating to eating food which a pig had also eaten and then suffering from diarrhea would need further investigation and could possibility be associated with transmission pathway for cysticercosis given Taenia solium is prevalent in pigs in many regions of Uganda?

**Response:** Insightful comment. While not mentioned in the interviews by name (we included only specifically named diseases), this is likely one of the pathogens.

**Comment:** It is interesting to note that bird flu (avian influenza) was mentioned once line 275 (zooanthroponoses) though respiratory diseases were included in table 2 therefore flu could be part of this specific zoonotic disease transmitted category. Another priority zoonosis is anthrax which was only mentioned in the discussion (line 395-396) of the paper associated with transmission from consumption of beef from sick cows. The One Health zoonotic disease prioritization for multisectoral engagement in Uganda (2017) included anthrax, zoonotic influenza viruses, viral haemorrhagic fevers, brucellosis, trypanosomiasis, plague and rabies. The Uganda One Health strategic plan 2018 -2022 had a goal to reduce the burden of prioritised zoonoses and AMR by 50 percent. The authors should discuss how their study may support Uganda to reduce the burden of prioritized zoonoses especially in this hotspot area.

**Response:** Revised accordingly both in introduction and conclusions.

**Comment:** In table 3 the demographic factors of rural, age and healthcare worker, were associated with overall zoonotic disease awareness. This table needs to define the “age” factor. From the OR = 0.40 it could be concluded that older participants are less aware of zoonotic diseases but given the small sample size it is important to include the confidence interval for this odds ratio result. It is logical to make the assumption that health care workers would have a significant greater zoonotic disease awareness given their training than other occupations (agriculture, livestock, farmers). It would be useful to further examine the ten health care workers education background and training.

**Response:** Confidence intervals added. Age categories were clarified.

**Comment:** The results description in lines 264-266 about proportion of participants with awareness of anthroponotic (animal- to- human) transmission mostly residing in rural setting (n=21/26) should be moved to the previous paragraph and it would be useful to know the occupations of these participants.

**Response:** Removed section as concept is covered by analysis (“Results from our multivariable model (see table 3) showed that people from rural areas were significantly more likely to be aware of zoonotic disease transmission compared to people who were from urban areas (OR = 3.98; P = 0.011).” Occupation is an interesting sub-analysis although we felt this would potentially bloat the results from this section.

**Comment:** The transmission knowledge of diseases from humans to animals were skewed toward transmission to gorillas (n=12/26). It is noted that one of the study team is Dr Benard Ssebide from the Gorilla Doctors and had been involved with the wildlife authority. It would be useful to review the interview guide to better understand the questions associated with zooanthroponosis knowledge and conduct further studies to evaluate the Uganda Wildlife Authority training for Park personnel and general public (noted in last paragraph of discussion). Did all 12 participants who knew about this risk transmission pathway from humans to gorilla’s undertake this training in the past and were most in the FGD men from Ruhija?

**Response:** The questions did not focus on zooanthroponosis as key area of focus. It emerged as we conducted interviews. We asked a single question about human to animal disease transmission under theme “diseases in animals” in the interview guide.

**Comment:** The study results about contact with animals is well presented and an important finding in this study. The sentence in line 298-299 requires changing to read more clearly and could be “There is recognition that disposal of animal remains requires contact with animals that can expose people to diseases”.

**Response:** Updated accordingly.

**Comment:** It is important to note that although some participants knew the animals could be infected with pathogens, they did not carry out appropriate infection prevention and biosecurity practices while handling carcasses. It would be useful to know if higher risk of zoonotic contact with animals was associated with gender? Is it mainly women who unset the animal traps? Gender was not found to be a demographic risk factor with overall zoonotic disease awareness as not included in table 3 results.

**Response:** This is a very interesting point but was not a key outcome in this study. Future research could definitely explore some of the gender differences around activities with high risk for spillover and accompanying beliefs.

**Comment:** The study results on contact with meat from sick and dead animals are important findings. The statement that “people eat the meat because of poverty” by village health teams of southern division demonstrates the need to understand the drivers of behaviour and addressing the root causes of risk taking. A One Health approach is needed to support community health through improving livestock and wildlife health. Training of Community animal health workers (CAHW’s) is needed and the World Organisation for Animal Health WOAH (formally OIE) has recently launched competency and curriculum guidelines for community animal health workers which should be referenced. <https://www.woah.org/app/uploads/2024/09/woah-competency-and-curriculum-guidelines-for-cahws-071024.pdf>. Additionally, the WOAH and African Union commenced a project called ZOOSURSY to further build One Health collaborations for disease surveillance of priority emerging and re-emerging zoonotic diseases with implementation of the WOAH Wildlife health framework. The reviewer recommends that outcomes from this small qualitative study may support further research in this zoonosis hotspot area and more widely.

**Response:** Great additional context. We have incorporated this accordingly into the manuscript.

**Comment:** In the reporting of disease outbreaks results the willingness of participants to report diseases in humans and animals was found to be low (n=28/153, 18%) with half of those reporting contacting the community health workers. As there was no mention of community animal health workers or veterinarians for reporting disease occurrence in animals – perhaps participants were more focused on disease outbreaks in humans?

Response: This does seem to be generally true. This is also consistent with the study finding that people would eat animals even if they were sick.

**Reviewer #3:**

**Comment:** Line 218, "diarrheal" should be "diarrhea".

**Response:** Corrected

**Comment:** Table 3: please list the age groups (i.e., <35 yrs, >35 yrs) in the table or table legend. Also, some cells are missing the percentages in parentheses.

Response: Corrected

**Comment:** Line 275: "influenza" not "Flue".

**Response:** Corrected

**Comment:** Line 291: the first sentence of this section ("Contact with animals") appears to be missing a word.

**Response:** Corrected

Comment: Line 294: a closed parenthesis is missing after "primates".

**Response:** Corrected

**CONCLUSIONS**

**Reviewer #1:**

**Comment:** My general feeling is that the authors have prioritised their quantitative results over the qualitative results – with the outcome being that the discussion and conclusion focus overly on a “lack of awareness” rather than the reasons for the lack of awareness, and the broader context around managing zoonotic disease risk in the community. This can be seen in the abstract, for example, where only the quantitative findings are mentioned. One of the benefits of using qualitative approaches alongside quantitative is that rather than just saying “awareness was low because XX% were not aware of X” – is that you can also start to think about why that might be the case, and what could be done to address this. The conclusion regarding lack of awareness is supported by the data, but I think there are additional interesting and worthwhile conclusions that could be drawn from the results (for example -the mention of poverty as a driver for activities which put individuals at risk of exposure to potentially infected meat). Situating “lack of awareness” as one of multiple challenges facing these communities would, I feel, give the results more weight and help in drawing some conclusions for how to advance research and action on zoonotic disease management.

**Response:** Updated accordingly. While our study does not explicitly address the potential link between awareness of health risks and subsequent behavior, it does, as reviewers have also pointed out, illustrate a key unexplored link in the transmission chain for zoonotic disease. Future explorations of the relationship, if any, between education, poverty, culture, and high-risk behaviors will be key in targeting effective interventions.

**Comment:** My concern really relates to the fact that the only recommendation of this paper is for policy makers and government to prioritise education regarding zoonotic diseases – it would be ideal if the authors could identify other recommendations on the basis of their results and to put these in a broader context. Additional reflections upon the limitations of the study would be appreciated.

**Response:** Updated accordingly.

**Reviewer #2:**

**Comment:** In the discussion several statements are missing references to support them – for example line 370 “our research augments previous studies into communities’ views of zoonotic diseases”.
The authors state that misconceptions about the origins of zoonotic diseases and transmission can be overcome through providing education citing a cross-sectional study in DRC following the end of the 2018 Ebola outbreak. The conclusion in this study recommended co-developed culturally sensitive and inclusive community evidence-based programs which compliments early anthropological insights from the West African Ebola epidemic about engaging communities and building trust. (Wilkinson A, Parker M, Martineau F, Leach M. 2017 Engaging ‘communities’: anthropological insights from the West African Ebola epidemic. Phil. Trans. R. Soc. B 372: 20160305. <http://dx.doi.org/10.1098/rstb.2016.0305>).
Increasing community knowledge and awareness alone is unlikely to be sufficient to successfully change human behaviours that drive zoonotic disease risk as described in a scoping review by Anna Durrance-Bagale et al (Drivers of zoonotic disease risk in the Indian subcontinent: A scoping review. One Health 2021. 13:100310). Co-designing context specific interventions using interdisciplinary and participatory methods are needed using One Health approaches. The cited (#31) Mangesho et al 2017 study among pastoralists in northern and eastern Tanzania also supports context specific interventions using One Health approaches taking into consideration social, cultural and economic aspects of communities.

**Response:** Very insightful points. We have updated this discussion section paragraph accordingly.

**Reviewer #3:** 

**Comment:** Lines 370-371: the sentence beginning, "While we found that only ⅓..." is a fragment.

**Response:** Corrected

**Comment:** Lines 371-373:: pastoralists were compared to what other group(s)? Please clarify.

Response: Corrected

**SUMMARY AND GENERAL COMMENTS**

**Reviewer #1:**

**Comment:** This is an interesting paper on an important topic, and the importance and the context of this topic was well established in the introduction. The strengths of this paper include the focus on a topic of high public health importance and inclusion of a community of direct relevance for the topic, and the broad range of topics included for discussion with the participants. This is already a good paper, but I would like to recommend some few changes which I think could help to improve this paper further. My requests for revision relate principally to the methodology, and the discussion/conclusion.

**Response:** Thank you for these useful comments, we have revised accordingly with special attention to the methodology and future implications mentioned in the discussion/conclusion sections.

**Comment:** I think the methodology could benefit from some additional structure and detail, and my comments on the method I have recommended use of the Standards for Reporting Qualitative Research (SRQR). My main concern relates to the mention of grounded theory in the abstract, which is not mentioned in the methodology – nor is any other approach to the qualitative data. Additional information on this would be needed. Additionally, somewhere in the paper (either in the introduction or the discussion) to understand the rationale behind why a mixed-methods approach was chosen.

**Response:** Revised accordingly.

**Comment:** I think the discussion could go further in putting the results in context and in the depth of analysis. For example, when observing that this study and many others have identified a lack of awareness – it may be useful to see if there are similarities which may contribute to the lack of awareness, or to consider the body of research that has suggested that awareness alone is not sufficient to change behavior. In addition to mentioning papers that have similar findings, it is important that the authors explain what broader conclusions or questions can be drawn from this, and thereby show the relevance of their research for broader contexts.

**Response:** Revised accordingly.

**Reviewer #2:**

**Comment:** A One Health approach with multi-sectoral and transdisciplinary collaborations, to zoonotic and reverse zoonosis prevention is urgently needed in this area as highlighted in this small semi-structured questionnaire FGD study. Currently strengthening of multisectoral collaboration for Mpox and anthrax prevention is occurring in the Kanungu district due to the location near DRC and cross-border interaction between school children. Training of approximately 200 school managers with IEC materials (posters and flyers) and a collaborative response action plan is centred on child-to-child strategy of empowering learners to share health message with peers, families and communities.
Further validation studies are required to evaluate the reason why some participants had awareness of reverse zoonosis transmission of disease from humans to gorillas. It is not evidence based to state that this reverse zoonosis awareness was due to targeted educational gorilla health programs surrounding BINP. A scoping review of all current educational programs and training relating to zoonosis and reverse zoonosis prevention and One Health practices is needed.

**Response:** Revised accordingly.

**Comment:** The recommendations need to be re-written in light of the reviewer’s comments. A One Health approach for co-developed culturally sensitive and inclusive community evidence-based programs for health system strengthening across human, animal/wildlife and ecosystem is needed. Additionally continuing to invest in long-term community livelihood projects that support poverty alleviation and improving the health of livestock and wildlife is recommended.

**Response:** Thank you also for your useful comments, we have revised accordingly with special attention paid to collaborative and transdisciplinary design.

**Reviewer #3:**

**Comment:** Overall, this is an interesting study reporting on the knowledge of rural and urban communities in southwestern Uganda on the possibility of transmission of infectious diseases between wild animals and humans. The manuscript is mostly well written, clear, and concise. Appropriate conclusions and policy recommendations have been made that are backed up by the findings.

PLOS authors have the option to publish the peer review history of their article ([what does this mean?](https://journals.plos.org/plosntds/s/editorial-and-peer-review-process#loc-peer-review-history)). If published, this will include your full peer review and any attached files.

**Response:** Thank you also for your useful comments, we have revised accordingly with special attention to the conclusions and recommendations.

We believe these revisions have significantly strengthened the manuscript and we hope it is now suitable for publication in *PLOS Neglected Tropical Diseases*. Thank you again for your guidance and for considering our revised manuscript.

Sincerely,

**Nahabwe Haven**

[**hahotice@googlemail.com**](mailto:hahotice@googlemail.com)
Corresponding Author
On behalf of all co-authors
