# Supplementary material for: Assessment of exposure to zoonoses and perceptions of zoonotic transmission surrounding the Bwindi impenetrable forest, Uganda
Source: PLoS Negl Trop Dis. 2025 Nov 17;19(11):e0013701. doi: 10.1371/journal.pntd.0013701 (PMC12633871; doi:10.1371/journal.pntd.0013701)
Supplement: Attachment — Submitted filename: Comments_PNTD-D-24-01469_R1.docx [file pntd.0013701.s007.docx]

### Overall comments

I thank the authors for the time they have taken to revise their manuscript in response to my comments and those of the other reviewers. I find the manuscript much improved, however, I still have significant concerns regarding the description of the methodology and overall research approach, which I have detailed below.

It is my sense from reading the revised manuscript that the authors need to rethink how they have described their approach. In particular, I think it needs to be indicated that this is a descriptive analysis – both for the qualitative and quantitative analysis.

Firstly, it seems to me that the authors have performed a purely descriptive analysis of their qualitative data. They do not really make use of their qualitative data in the results/discussion beyond a description used to support the quantitative results, and so are not really giving us an in-depth insight into the perspectives of the participants. As mentioned in my previous comments, if the authors have applied grounded theory, I would expect some further explanation of how this was actually applied – from what is currently written, the approach seems to me more aligned with something like content analysis than grounded theory (most notably because the authors have not developed a theory from the data), and the focus seems to be on description rather than “richer understanding” (line 215)... but I am less interested in the authors applying a particular label to their work than I am about having a clear and transparent explanation of what was actually done with the data.

To give an example – the verbatim in lines 280-281 gives an example of where a participant described disease transmission through sharing food, supporting an explanation in lines 278-279. But it is not being used to construct an argument or a narrative around what participants believe – it is purely descriptive. Using qualitative data to make a purely descriptive analysis is perfectly ok! But it does not seem compatible with the methodology as currently described by the authors in this paper.

Secondly, it is still not clear to me what the source of the quantitative data is – the only sentence given in the methods is “scoring and ranking data obtained from the focus group discusses were analysed using descriptive and non-parametric statistical methods” (lines 236-237). What was the raw dataset used for this analysis? It is my understanding that a semi-structured interview guide was used to collect data during the focus groups. Was there also a questionnaire of some sort that was used to collect quantitative data? Or were the transcripts used as a base for building quantitative data, in which case how was this done? This is extremely important to clarify as it is not transparent how the authors came to the conclusions described in their results.

For example, the authors say “Only 61 of 153 (33%) participants perceived that animals could transmit diseases to humans” (lines 271-279). On what basis did they make this judgement? Were there specific questions designed to assess this? Or was this based on analysis of the transcripts from the focus group discussions, in which case what were the criteria used to determine if participants knew/perceived about zoonotic disease transmission?

So, I would suggest the following changes to address these general concerns:

1. **For the objective:** “Using a mixed methods approach, the goal of this study was to better understand communities’ contacts with wild and domestic animals and their knowledge of potential disease risks associated with these interactions.” – change the word “understand” to “describe” to reflect the fact that this is a descriptive study.
2. **For the methodology:** Be more explicit about what data was used for the qualitative data analysis vs. the quantitative data analysis, and what type of analysis was performed. This concerns the “mixed methods approach”, and “data analysis” sections. As it stands, I am not convinced that grounded theory was used (though I am happy to be proved wrong, if the authors dispute this). It seems more to me that a qualitative descriptive analysis was performed, something along the lines of **content analysis**. This would fit better with the idea of descriptive verbatims and frequency-counts. But again, I am less interested in the label applied to the analysis than having a clear and transparent explanation of it. I strongly encourage the authors to look at the following papers when thinking about how to revise the presentation of their analytic approach:
   a. Vaismoradi, M., Turunen, H., Bondas, T., 2013. Content analysis and thematic analysis: Implications for conducting a qualitative descriptive study. Nursing & Health Sciences 15, 398–405. <https://doi.org/10.1111/nhs.12048>
   b. Cho, J., Lee, E.-H., 2014. Reducing Confusion about Grounded Theory and Qualitative Content Analysis: Similarities and Differences. The Qualitative Report 19, 1–20. <https://doi.org/10.46743/2160-3715/2014.1028>

Based on the comments I have provided, the authors also need to revise their abstract to reflect the changes made (see in my detailed comments below).

### Abstract

The information in the manuscript itself and in the abstract needs to be aligned to reflect the changes made in the manuscript.

**Methods section in abstract:**

My comment on the “three-stage coding framework” which is mentioned in the abstract but not at all in the data analysis has still not been addressed. Either the authors need to explain what is meant by a three-stage coding framework in the data analysis section, or this should be removed from the abstract.

A questionnaire is mentioned in the abstract, but not in the results (where it is said data was collected using an interview guide). Was a questionnaire (for quantitative results) also used? This causes confusion about the nature of data collection. Please see my comments below for further information.

**Results section in abstract:**

It seems like the authors have pulled out just two of the findings of their study for the “results” section. They don’t mention the themes they identified, or the *overall* results of their study. It is still not clear to me what is considered a “theme” (see my comments below), but for the qualitative aspect the themes should be indicated in the abstract, and for the quantitative work there should be an *overview* of the quantitative results (not just noting one OR).

### Methodology

**General structure**

The current ordering of the sections seems a bit counterintuitive to me. I would suggest the following order for the methods section for clarity:

1. Mixed-methods approach
2. Study area and context
3. Participants and sampling strategy
4. Data collection
5. Data analysis

**Mixed methods approach**

Please see my comments above about the importance of clearly articulating the approach used for both quantitative and qualitative analysis.

The link between the methods chosen and the research question/context should be better justified – for example, why does a “lack of prior research” mean a mixed-methods approach is needed? Doesn’t that justify research of any nature? What is the added value of a mixed-method approach here? What I am really trying to get at is an understanding of *why* the authors didn’t do just qualitative or just quantitative research alone.

This section should also present clearly the approach used for both qualitative and for quantitative methods.

**Data collection (Lines 163-175):**

I appreciate that the authors have created a new section called “Data collection” as I suggested – however, they also need to explain the *method* that was used for data collection. I understood that focus groups were used from elsewhere in the manuscript but this is not mentioned anywhere in this section. The authors should indicate that focus groups were used, and I would recommend moving lines 188-190 to the data collection section rather than the participants section.

Is it possible to include a copy of the English-language interview guide in the supplementary material? I think this could help resolve a lot of the questions I have about what data was collected and analysed.

**Participants (lines 176-197)**

I recommend this be called “Participants and sampling strategy”

From the revisions, I understand further that the sampling was purposive, based on the description given by the authors, with the consequence that the sample is not representative. This is not a problem in and of itself – in fact, a non-representative sample is perfectly reasonable and in line with how much of qualitative research is conducted – however, conclusions should not be extrapolated based on the quantitative findings to the rest of the population. The authors could add a sentence on this in the discussion to make this point clear and should be careful about wording in the results (see my comments below).

I am not sure why the authors added a reference to saturation in their method (lines 197) in their revision of the article. Saturation is a very specific and theoretically-bounded concept, which is not appropriate in all circumstances. If the authors do want to make reference to saturation, it would be important to define what they understand saturation to mean, and *why* this was judged the most appropriate method to determine final sample size for this study with reference to the research aim.

**Data analysis (Lines 163-175):**

In line with my comments above, there needs to be greater explanation of the analytic method used. There are a lot of general qualitative terms used without explanation, and I have questions remaining based on the revised material.

- For: “All transcribed text was analyzed using grounded theory independently by four researchers (HN, JJ, JL and SK)”. What does it mean for four people to apply grounded theory separately? Again, I am not sure that this is what was done… but if it was, how were four separate theories integrated together?
- For: “First, researchers read through who participated in the data and began to develop codes and concepts within the text. Next, they created themes and sub-themes such as medical treatments or disease symptoms.” – on the basis of what? the frequency with which the topics were mentioned? the importance of the topic as judged by the researchers? How do the authors define a code, and a theme? How were the codes from different researchers brought together into themes?
- This is the first and only time that “conceptual frameworks” are mentioned. What do the authors consider to be a conceptual framework and where is it visible in their results?

### Results

**General comment:** As mentioned above under sampling, this is not a representative population. That’s fine for the purposes of a descriptive analysis, but requires some care with regards to the conclusions drawn on the basis of the quantitative results. This is mostly ok in the results, but some cases this could be clearer. For example – for lines 317-323 I would replace “people” with “participants” in all sentences to be clear that the OR refer to the participants and not the general population.

**Themes and Table 4:** It is not clear to me what the authors consider a theme (see my comments above on data analysis). I had assumed that the subheadings of the results were intended to be the themes (as is commonly done in many qualitative papers) – but after the addition of Table 4 in this revision, I see this is not the case. What is the relationship between the subheadings of the results and the themes? This should be clarified. In addition, I am not totally sure of the purpose of Table 4. As it currently stands, this table is not properly integrated in the results text. There is only one reference made to it in the text (line 330). Most of the items in this table are not discussed at all.

**Line 324:** I am uncomfortable with the heading that has been added in this revision “Perception of Risk of Zooanthroponotic Diseases” as being placed in contradistinction to “Perception of Risk of Zoonotic Diseases”. I understand zoonoses are defined differently depending on the source of the definition, but it is sufficiently commonly accepted to consider zoonotic diseases to include disease spread between humans and animal in both directions, that if the authors are using another definition this should be explicitly specified somewhere in the results.

**Line 246:** Somewhere before going into the details of the characteristics of the participants it should be mentioned how many participants total there were (153 from my reading of the results).

**Tables 2 and 3:** It would be nice to have a some more narrative around these two tables either in the results or the discussion. I note that the only result that is pulled out for the discussion (and the abstract) is the OR related to overall zoonotic awareness being greater in rural rather than urban populations from Table 2. The other ORs in this table are not significant but they could still be discussed – for example, the fact that these other ORs are all quite close to 1 and don’t all go in the same direction (i.e. sometimes rural, sometimes urban have better awareness) – why do the authors think this might be the case? Again, this needs to be contextualised with a better understanding of how the authors determined what was awareness vs. not awareness based on the results (to be described in the methods), as that would also have an important impact on the findings, and might aid in their interpretation.

Table 3 is based on demographic information, so it is clear how the two groups were identified – but nonetheless, some comments on these results from the authors would be appreciated.

### Discussion/conclusion

I think the sentence added to the conclusion (lines 498-500) is quite general and does not relate to the results of the paper (or the sentences immediately before it). I think it would make much more sense to talk about the importance of this information for building contextualised and localised approaches to zoonotic disease prevention and management. The authors wrote very nicely about this in lines 480-489 in the discussion in response to a comment from reviewer 2. To me the real value of this study is that it provides a description of a population that is understudied and may have specific and contextual challenges that are not addressed by general national/international policy. This information is very useful and important in helping to inform local strategies for disease management etc.

I do find it a bit disappointing that the overall conclusion remains to “prioritise education”, and that the overall slant of the discussion is focused on the “lack of awareness” of participants – I don’t think my comments in the original review on this point have been addressed, so I will provide some further detail on this point.

The idea of “improving education” or “lack of awareness” is a very general conclusion/approach often taken in research undertaken in LMICs (though not only), and seems to ignore the fact that improving knowledge does not always lead to changes in behaviour, or that individuals may already have sufficient knowledge, but are not engaging in specific behaviours because they have other reasons or priorities. One of the key benefits of studies like this is that it allows us to identify what those reasons/priorities might be.

For example, it is mentioned in the results that “Meat is highly prized and limited, particularly since hunting is prohibited in the BINP” (lines 382-383), and reasons for eating meat are “extreme hunger due to poverty and potential economic loss” (lines 399-400). This means that individuals may well be very aware that they are at risk of disease transmission if eating meat from sick animals, but do so anyway due to the value of meat or lack of available food. In such a case, it is hard to say how further education or awareness raising would resolve this situation. I would have thought that results such as this point to the intractable and systemic nature of problems related to the specific context of the study, but which may have echoes in other settings – and therefore point to the need for a more contextualised policy approach which takes systemic factors into account, or to include field actors to a greater extent in the construction of policy.

I note that reviewer 2 also made similar comments on the discussion – I would encourage the authors to think more deeply about how their findings could be used to inform the development and implementation of locally-relevant diseases management/mitigation policies, the importance of engaging with field actors, and the feasibility of implementing strategies for change in communities which face intractable problems such as poverty and poor access to health services. The authors do not have to come up with the solutions to these very challenging problems! But they do need to openly acknowledge the impact such factors have on disease risk mitigation and provide some thought on their impact for making use of their results. This paper has a lot of useful information and it would be a shame to suggest the only conclusion it has to offer is to improve education.
