# Supplementary material for: Assessment of exposure to zoonoses and perceptions of zoonotic transmission surrounding the Bwindi impenetrable forest, Uganda
Source: PLoS Negl Trop Dis. 2025 Nov 17;19(11):e0013701. doi: 10.1371/journal.pntd.0013701 (PMC12633871; doi:10.1371/journal.pntd.0013701)
Supplement: Attachment — Submitted filename: Zoonosis PLOS NTD PNTD-D-24-01469_13OCT_Reviewer Response.docx [file pntd.0013701.s008.docx]

**NAHABWE Haven**

**Bwindi Community Hospital,**

**P.O BOX 58, Kanungu, Uganda**

[hahotice@googlemail.com](mailto:hahotice@googlemail.com)

Dear PLOS Neglected Tropical Diseases Editorial Team,

**Re: Manuscript ID PNTD-D-24-01469**
**Title: “Assessment of exposure to zoonoses and perceptions of zoonotic transmission surrounding the Bwindi Impenetrable Forest, Uganda”**

Thank you for the opportunity to revise our manuscript. We appreciate the constructive comments and suggestions provided by you and the reviewers, which have helped us to improve the clarity and quality of our work. We have carefully considered each point raised and revised the manuscript accordingly.

**Additional Editor Comments:**

Reviewers raised serious methodological concerns regarding the approach used in this study, as well as issues related to previously provided comments that remain unaddressed. We therefore invite the authors to revise their manuscript, taking these concerns into account, and to provide a detailed point-by-point response to all reviewer comments.
For the quantitative component, the authors should clearly describe the data used in the analysis, explain how the outcome variable (zoonotic understanding) was derived, and provide more detail on the analytical methods and results, including how model performance was assessed. I'm not sure what you meant by "while minimizing the Akaike Information Criterion (AIC)." Do you mean that you selected the model with the lowest AIC value?

**Response: We have provided detailed explanation to this in the revision.**


**Journal Requirements:**

1) Please ensure that the CRediT author contributions listed for every co-author are completed accurately and in full.
At this stage, the following Authors/Authors require contributions: Scott Kellermann, Evan Andrew Rusoja, Micheal Wilkes, Nicole R Gardner, and Tierra Smiley Evans. Please ensure that the full contributions of each author are acknowledged in the "Add/Edit/Remove Authors" section of our submission form.The list of CRediT author contributions may be found here: https://journals.plos.org/plosntds/s/authorship#loc-author-contributions

**Response:** This has been addressed as requested.

2) We have noticed that you referred to "Supplementary material Appendix 1" on page 5 in your manuscript. However, there is no corresponding file uploaded to the submission. Please upload it as a separate file with the item type 'Supporting Information'. Please add a full list of legends for your Supporting Information files after the references list.

**Response:** This has been provided with attachment name "Supplementary material Appendix 1

3) Please ensure that the Data Availability Statement mentioned in the manuscript matches the one provided in the online submission form.

**Response:** This section has been edited to match the one provided in the online submission form

Note: If the reviewer comments include a recommendation to cite specific previously published works, please review and evaluate these publications to determine whether they are relevant and should be cited. There is no requirement to cite these works unless the editor has indicated otherwise.

**Comments to the Authors:**

**Please note that one of the reviews is uploaded as an attachment.**

**Reviewers' Comments:**

Reviewer's Responses to Questions

**Key Review Criteria Required for Acceptance?**
As you describe the new analyses required for acceptance, please consider the following:

**Methods**

-Are the objectives of the study clearly articulated with a clear testable hypothesis stated?

-Is the study design appropriate to address the stated objectives?

-Is the population clearly described and appropriate for the hypothesis being tested?
-Is the sample size sufficient to ensure adequate power to address the hypothesis being tested?
-Were correct statistical analysis used to support conclusions?
-Are there concerns about ethical or regulatory requirements being met?

Reviewer #1: Please see the attached document

Reviewer #2: The title of the manuscript could be more descriptive of the study. The reviewer suggests a title including “Study of zoonoses and anthropozoonosis knowledge and perception of transmission activities in rural and urban tribal communities surrounding the Bwindi Impenetrable Forest in Uganda”.

**Response:** Upon consultation with the rest of the study team members, we felt that the title we have was an appropriate study. We have provided more details to make the study better for this title as well.

The mixed-methods study methodology section is much clearer. The reviewer acknowledges updates:
•No participants withdraw from the study line
•Inclusion of study being conducted near the Bwindi Impenetrable Forest in Uganda
•Importance of One Health approach in zoonotic disease control

Reviewer #4: Overall, the results presented match the goals of the analysis. Here are some few recommendations: I will advise authors to consider some potential limitations of the snowball technique used. Include some limitations of using this sampling method in your discussion. As it is non randomized, what are the potential limitations that would impact your data and results? This was not discussed.

**Response:** A paragraph has been added to highlight this.

Line 171 - was this selection done before the snowball sampling was done? If after, was sample size sufficient for the investigation? You stated that target population was individuals with direct or indirect contact with wild animals, what were the other group/s excluded and was there a reason? Please make this clear

**Response:** Our study area was in specified geographical areas. Urban or rural areas. The main criteria used that participants were 18 years old or greater and had resided in the community for at least five years. For this study, 5 years was seen as enough time to have adequate knowledge about a participant’s community. There was error when proposed that prferredntial selection had been. The statement has been revised to read as “In rural communities, because sampling was done in communities adjacent to BINP, participants were most like to have direct or indirect wildlife.”

**Results**

-Does the analysis presented match the analysis plan?
-Are the results clearly and completely presented?
-Are the figures (Tables, Images) of sufficient quality for clarity?

Reviewer #1: Please see the attached document

Reviewer #2: The aim of the study was to better understand communities contacts with wild and domestic animals and their knowledge of potential disease risks associated with interactions around the Bwindi Impenetrable National Park, a known foci for spillover events.
Knowledge is skills and information which is objective while awareness or perception is interchangeable and is experiences of the observer which helps a person understand better. Knowledge is used when the information is factual like figure 2 summarizes community knowledge about transmission. Awareness is associated with perceptions people have and exhibit.
It is still unclear to the reviewer how overall zoonotic awareness in Table 2 has been calculated.

Reviewer #4: Figure 2- figure captions should be at the bottom.

**Response:** This has been changed.

Table 2- state if this is a univariable analysis. Parenthesis missing in contact with vermins. – **Response:** Parenthesis has been added.

Table 3 and line 289, if you want to include age as a factor, I will advise you flip this round and use <35 as associated, and put the OR, instead of >35 which does not show association in the tables.

**Response:** We have reviewed this and we feel that the way we had presented data makes sense. The interpretation we have is that “Age (>35) has an OR of 0.40 with a 95% CI of (0.19–0.84). The p-value is 0.02. Since the 95% CI does not include 1.0 and the P-Value (0.02) is less than 0.05, there is a statistically significant association.” Hopefully this gives you and idea on how we are interpreting this particular factor.

**Conclusions**

-Are the conclusions supported by the data presented?

-Are the limitations of analysis clearly described?

-Do the authors discuss how these data can be helpful to advance our understanding of the topic under study?

-Is public health relevance addressed?

Reviewer #1: Please see the attached document

Reviewer #2: The reviewer acknowledges the significantly improved conclusion emphasising the need for One Health strategies at national and international levels.

Reviewer #4: Line 393 – “We found that only 1/3 of the respondents were aware of zoonotic diseases, with rural communities having higher awareness” what do you suggest could explain this? If your data cannot explain this, would you suggest that future research is needed to understand this….?

**Response:** We have provided an explanation for this observation.

Line 419- I understand that this maybe beyond the scope of this study, however, it does come up in your MLR that rural has more awareness, you need to provide at least some reasons why you believe this is so and then advice that further research can be done….
**Response:** We have provided an explanation for this observation.

Line 448 - It will be good to include any suggestions here on the type of study that will be relevant

**Response:** We have provided suggestions on areas/types of studies to be included.

**Editorial and Data Presentation Modifications?**


Use this section for editorial suggestions as well as relatively minor modifications of existing data that would enhance clarity. If the only modifications needed are minor and/or editorial, you may wish to recommend “Minor Revision” or “Accept”.

Reviewer #1: Please see the attached document

Reviewer #2: (No Response)

Reviewer #4: I have recommended some modifications that I believe will enhance the clarity of this publication. I will recommend minor revisions

**Response:** We have tried in the best way possible to address the concerns/comments raised.

**Summary and General Comments**

Use this section to provide overall comments, discuss strengths/weaknesses of the study, novelty, significance, general execution and scholarship. You may also include additional comments for the author, including concerns about dual publication, research ethics, or publication ethics. If requesting major revision, please articulate the new experiments that are needed.

Reviewer #1: Please see the attached document

Reviewer #2: The revised manuscript is much improved and most reviewer comments were addressed.

Reviewer #4: Overall, the goals of the study was answered however, I have given a few points that the authors should consider. “the goal of this study was to better understand communities’ contacts with wild and domestic animals and their knowledge of potential disease risks associated with these interactions”. Add if this for policy purposes or for awareness raising somewhere in the discussion.
Some strengths of the research includes that the translation was reviewed by research team who were fluent in the local languages to ensure that meaning of words in the questionnaire was retained.

The methodology used, mixed model was appropriate, and odds ratio including confidence intervals were reported, I have advised risk factors such as decreasing age be reported rather than increasing age which seem like a protective factor.

It is an important piece of research that helps to provide some information on the understanding of zoonoses in the study area. I recommend for publication after revision of the minor corrections.

**Response:** We have provided more information to address the comments raised. We hope these have addressed the comments raised.

PLOS authors have the option to publish the peer review history of their article ([what does this mean?](https://journals.plos.org/plosntds/s/editorial-and-peer-review-process#loc-peer-review-history)). If published, this will include your full peer review and any attached files.

If you choose “no”, your identity will remain anonymous but your review may still be made public.

**Do you want your identity to be public for this peer review?** For information about this choice, including consent withdrawal, please see our [Privacy Policy](https://www.plos.org/privacy-policy).

Reviewer #1: No

Reviewer #2: **Yes:** Andrea Britton

Reviewer #4: **Yes:** Bibiana Zirra Shallangwa

[NOTE: If reviewer comments were submitted as an attachment file, they will be attached to this email and accessible via the submission site. Please log into your account, locate the manuscript record, and check for the action link "View Attachments". If this link does not appear, there are no attachment files.]

**Figure resubmission:**

While revising your submission, please upload your figure files to the Preflight Analysis and Conversion Engine (PACE) digital diagnostic tool, https://pacev2.apexcovantage.com/. PACE helps ensure that figures meet PLOS requirements. To use PACE, you must first register as a user. Registration is free. Then, login and navigate to the UPLOAD tab, where you will find detailed instructions on how to use the tool. If you encounter any issues or have any questions when using PACE, please email PLOS at figures@plos.org. Please note that Supporting Information files do not need this step. If there are other versions of figure files still present in your submission file inventory at resubmission, please replace them with the PACE-processed versions.

**Response:** This has been done and new figures have been resubmitted.

**Reproducibility:**

To enhance the reproducibility of your results, we recommend that authors of applicable studies deposit laboratory protocols in protocols.io, where a protocol can be assigned its own identifier (DOI) such that it can be cited independently in the future. Additionally, PLOS ONE offers an option to publish peer-reviewed clinical study protocols. Read more information on sharing protocols at <https://plos.org/protocols?utm_medium=editorial-email&utm_source=authorletters&utm_campaign=protocols>

**Response:** We have provided more information to the study. There were no laboratory protocols used for this study apart from an interview guide which we have provided.

**DETAILED RESPONSE TO REVIEWER 1 COMMENTS**

### Overall comments

I thank the authors for the time they have taken to revise their manuscript in response to my comments and those of the other reviewers. I find the manuscript much improved; however, I still have significant concerns regarding the description of the methodology and overall research approach, which I have detailed below.

It is my sense from reading the revised manuscript that the authors need to rethink how they have described their approach. In particular, I think it needs to be indicated that this is a descriptive analysis – both for the qualitative and quantitative analysis.

Firstly, it seems to me that the authors have performed a purely descriptive analysis of their qualitative data. They do not really make use of their qualitative data in the results/discussion beyond a description used to support the quantitative results, and so are not really giving us an in-depth insight into the perspectives of the participants. As mentioned in my previous comments, if the authors have applied grounded theory, I would expect some further explanation of how this was actually applied – from what is currently written, the approach seems to me more aligned with something like content analysis than grounded theory (most notably because the authors have not developed a theory from the data), and the focus seems to be on description rather than “richer understanding” (line 215)... but I am less interested in the author’s applying a particular label to their work than I am about having a clear and transparent explanation of what was actually done with the data.

After careful review, and reviewing the materials, it seems we actually conducted thematic analysis. We have changed this to this effect and provided as much detail as possible.

To give an example – the verbatim in lines 280-281 gives an example of where a participant described disease transmission through sharing food, supporting an explanation in lines 278-279. But it is not being used to construct an argument or a narrative around what participants believe – it is purely descriptive. Using qualitative data to make a purely descriptive analysis is perfectly ok! But it does not seem compatible with the methodology as currently described by the authors in this paper.

**Response:** We have tried to address the concerns raised. We have provided more detail to make our submission stronger.

Secondly, it is still not clear to me what the source of the quantitative data is – the only sentence given in the methods is “scoring and ranking data obtained from the focus group discusses were analysed using descriptive and non-parametric statistical methods” (lines 236-237). What was the raw dataset used for this analysis? It is my understanding that a semi-structured interview guide was used to collect data during the focus groups. Was there also a questionnaire of some sort that was used to collect quantitative data? Or were the transcripts used as a base for building quantitative data, in which case how was this done? This is extremely important to clarify as it is not transparent how the authors came to the conclusions described in their results.

**Response:** We have added more details on how quantitative data was obtained.

For example, the authors say “Only 61 of 153 (33%) participants perceived that animals could transmit diseases to humans” (lines 271-279). On what basis did they make this judgement? Were there specific questions designed to assess this? Or was this based on analysis of the transcripts from the focus group discussions, in which case what were the criteria used to determine if participants knew/perceived about zoonotic disease transmission?

**Response:** This more like 33% were aware that animals can transmit diseases to humans. We have changed this since “perception” differs from awareness.

So, I would suggest the following changes to address these general concerns:

1. **For the objective:** “Using a mixed methods approach, the goal of this study was to better understand communities’ contacts with wild and domestic animals and their knowledge of potential disease risks associated with these interactions.” – change the word “understand” to “describe” to reflect the fact that this is a descriptive study.

**Response:** This has been updated.

2. **For the methodology:** Be more explicit about what data was used for the qualitative data analysis vs. the quantitative data analysis, and what type of analysis was performed. This concerns the “mixed methods approach”, and “data analysis” sections. As it stands, I am not convinced that grounded theory was used (though I am happy to be proved wrong, if the authors dispute this). It seems more to me that a qualitative descriptive analysis was performed, something along the lines of **content analysis**. This would fit better with the idea of descriptive verbatims and frequency-counts. But again, I am less interested in the label applied to the analysis than having a clear and transparent explanation of it. I strongly encourage the authors to look at the following papers when thinking about how to revise the presentation of their analytic approach:
   a. Vaismoradi, M., Turunen, H., Bondas, T., 2013. Content analysis and thematic analysis: Implications for conducting a qualitative descriptive study. Nursing & Health Sciences 15, 398–405. <https://doi.org/10.1111/nhs.12048>
   b. Cho, J., Lee, E.-H., 2014. Reducing Confusion about Grounded Theory and Qualitative Content Analysis: Similarities and Differences. The Qualitative Report 19, 1–20. <https://doi.org/10.46743/2160-3715/2014.1028>

**Response:** Updated accordingly.

Based on the comments I have provided, the authors also need to revise their abstract to reflect the changes made (see in my detailed comments below).

### Abstract

The information in the manuscript itself and in the abstract needs to be aligned to reflect the changes made in the manuscript.

**Methods section in abstract:**

My comment on the “three-stage coding framework” which is mentioned in the abstract but not at all in the data analysis has still not been addressed. Either the authors need to explain what is meant by a three-stage coding framework in the data analysis section, or this should be removed from the abstract.

**Response:** Updated to reflect the above change.

A questionnaire is mentioned in the abstract, but not in the results (where it is said data was collected using an interview guide). Was a questionnaire (for quantitative results) also used? This causes confusion about the nature of data collection. Please see my comments below for further information.

**Response:** These have been revised. An interview guide has also been provided.

**Results section in abstract:**

It seems like the authors have pulled out just two of the findings of their study for the “results” section. They don’t mention the themes they identified, or the *overall* results of their study. It is still not clear to me what is considered a “theme” (see my comments below), but for the qualitative aspect the themes should be indicated in the abstract, and for the quantitative work there should be an *overview* of the quantitative results (not just noting one OR).

**Response:** Updated to be more inclusive of the broader findings.

### Methodology

**General structure**

The current ordering of the sections seems a bit counterintuitive to me. I would suggest the following order for the methods section for clarity:

1. Mixed-methods approach
2. Study area and context
3. Participants and sampling strategy
4. Data collection
5. Data analysis

**Response:** Sections updated accordingly.

**Mixed methods approach**

Please see my comments above about the importance of clearly articulating the approach used for both quantitative and qualitative analysis.

The link between the methods chosen and the research question/context should be better justified – for example, why does a “lack of prior research” mean a mixed-methods approach is needed? Doesn’t that justify research of any nature? What is the added value of a mixed-method approach here? What I am really trying to get at is an understanding of *why* the authors didn’t do just qualitative or just quantitative research alone.

This section should also present clearly the approach used for both qualitative and for quantitative methods.

**Response:** Revised and updated.

**Data collection (Lines 163-175):**

I appreciate that the authors have created a new section called “Data collection” as I suggested – however, they also need to explain the *method* that was used for data collection. I understood that focus groups were used from elsewhere in the manuscript but this is not mentioned anywhere in this section. The authors should indicate that focus groups were used, and I would recommend moving lines 188-190 to the data collection section rather than the participants section.

Is it possible to include a copy of the English-language interview guide in the supplementary material? I think this could help resolve a lot of the questions I have about what data was collected and analysed.

**Response:** A copy of the English guide has been provided as supplemental information.

**Participants (lines 176-197)**

I recommend this be called “Participants and sampling strategy”

From the revisions, I understand further that the sampling was purposive, based on the description given by the authors, with the consequence that the sample is not representative. This is not a problem in and of itself – in fact, a non-representative sample is perfectly reasonable and in line with how much of qualitative research is conducted – however, conclusions should not be extrapolated based on the quantitative findings to the rest of the population. The authors could add a sentence on this in the discussion to make this point clear and should be careful about wording in the results (see my comments below).

I am not sure why the authors added a reference to saturation in their method (lines 197) in their revision of the article. Saturation is a very specific and theoretically-bounded concept, which is not appropriate in all circumstances. If the authors do want to make reference to saturation, it would be important to define what they understand saturation to mean, and *why* this was judged the most appropriate method to determine final sample size for this study with reference to the research aim.

**Response:** For this study, data collection ceased upon reaching saturation, which was defined as the point where no new data or categories emerged from participant interviews. This is consistent with the sampling method and the explorative nature of this study, however, the point about limitations is very well taken and has been updated accordingly in the sections suggested. *Hennink, Monique, and Bonnie N. Kaiser. "Sample sizes for saturation in qualitative research: A systematic review of empirical tests." Social science & medicine 292 (2022): 114523.*

**Data analysis (Lines 163-175):**

In line with my comments above, there needs to be greater explanation of the analytic method used. There are a lot of general qualitative terms used without explanation, and I have questions remaining based on the revised material.

- For: “All transcribed text was analyzed using grounded theory independently by four researchers (HN, JJ, JL and SK)”. What does it mean for four people to apply grounded theory separately? Again, I am not sure that this is what was done… but if it was, how were four separate theories integrated together?

**Response:** During data analysis, all transcribed interviews were subjected to thematic analysis to identify and organize patterns of meaning related to the study objectives. This study adopted a hybrid approach to data analysis, beginning with inductive coding on a few transcripts and then the resulting codebook was applied deductively to the remainder. At first, two researchers (HN and JJ) separately and inductively coded a preliminary subset of interviews (two transcripts each) and then then participated in a consensus meeting to compare their initial codes and to derive concepts. Through discussion, final preliminary codebook to use for the full analysis was developed.

- For: “First, researchers read through who participated in the data and began to develop codes and concepts within the text. Next, they created themes and sub-themes such as medical treatments or disease symptoms.” – on the basis of what? the frequency with which the topics were mentioned? the importance of the topic as judged by the researchers? How do the authors define a code, and a theme? How were the codes from different researchers brought together into themes?

**Response:** For this study, codes were defined as short, descriptive labels assigned to meaningful units of text while themes were broader, conceptual patterns that combine multiple related codes. Several main themes were agreed upon by the research team based on their relevance to the overall aims of the project and are described in Table 4. There were continuous meetings to ensure that application of the codebook was consistent and reliability for all the four doing analysis.

This is the first and only time that “conceptual frameworks” are mentioned. What do the authors consider to be a conceptual framework and where is it visible in their results?

**Response:** Revised for more accurate description.

### Results

**General comment:** As mentioned above under sampling, this is not a representative population. That’s fine for the purposes of a descriptive analysis, but requires some care with regards to the conclusions drawn on the basis of the quantitative results. This is mostly ok in the results, but some cases this could be clearer. For example – for lines 317-323 I would replace “people” with “participants” in all sentences to be clear that the OR refer to the participants and not the general population.

**Response:** This has been revised.

**Themes and Table 4:** It is not clear to me what the authors consider a theme (see my comments above on data analysis). I had assumed that the subheadings of the results were intended to be the themes (as is commonly done in many qualitative papers) – but after the addition of Table 4 in this revision, I see this is not the case. What is the relationship between the subheadings of the results and the themes? This should be clarified. In addition, I am not totally sure of the purpose of Table 4. As it currently stands, this table is not properly integrated in the results text. There is only one reference made to it in the text (line 330). Most of the items in this table are not discussed at all.

**Response:** Revised to summarize the themes.

**Line 324:** I am uncomfortable with the heading that has been added in this revision “Perception of Risk of Zooanthroponotic Diseases” as being placed in contradistinction to “Perception of Risk of Zoonotic Diseases”. I understand zoonoses are defined differently depending on the source of the definition, but it is sufficiently commonly accepted to consider zoonotic diseases to include disease spread between humans and animal in both directions, that if the authors are using another definition this should be explicitly specified somewhere in the results.

**Response:** We consolidated the sections for simplicity and clarity.

**Line 246:** Somewhere before going into the details of the characteristics of the participants it should be mentioned how many participants total there were (153 from my reading of the results).

**Response:** This has been added.

**Tables 2 and 3:** It would be nice to have a some more narrative around these two tables either in the results or the discussion. I note that the only result that is pulled out for the discussion (and the abstract) is the OR related to overall zoonotic awareness being greater in rural rather than urban populations from Table 2. The other ORs in this table are not significant but they could still be discussed – for example, the fact that these other ORs are all quite close to 1 and don’t all go in the same direction (i.e. sometimes rural, sometimes urban have better awareness) – why do the authors think this might be the case? Again, this needs to be contextualised with a better understanding of how the authors determined what was awareness vs. not awareness based on the results (to be described in the methods), as that would also have an important impact on the findings, and might aid in their interpretation.

**Response:** We’ve made some revisions to these sections although some of these areas remain important targets for future investigation.

Table 3 is based on demographic information, so it is clear how the two groups were identified – but nonetheless, some comments on these results from the authors would be appreciated.

**Response:** Brief conversation included below table and better integrated into future discussion.

### Discussion/conclusion

I think the sentence added to the conclusion (lines 498-500) is quite general and does not relate to the results of the paper (or the sentences immediately before it). I think it would make much more sense to talk about the importance of this information for building contextualised and localised approaches to zoonotic disease prevention and management. The authors wrote very nicely about this in lines 480-489 in the discussion in response to a comment from reviewer 2. To me the real value of this study is that it provides a description of a population that is understudied and may have specific and contextual challenges that are not addressed by general national/international policy. This information is very useful and important in helping to inform local strategies for disease management etc.

I do find it a bit disappointing that the overall conclusion remains to “prioritise education”, and that the overall slant of the discussion is focused on the “lack of awareness” of participants – I don’t think my comments in the original review on this point have been addressed, so I will provide some further detail on this point.

The idea of “improving education” or “lack of awareness” is a very general conclusion/approach often taken in research undertaken in LMICs (though not only), and seems to ignore the fact that improving knowledge does not always lead to changes in behaviour, or that individuals may already have sufficient knowledge, but are not engaging in specific behaviours because they have other reasons or priorities. One of the key benefits of studies like this is that it allows us to identify what those reasons/priorities might be.

For example, it is mentioned in the results that “Meat is highly prized and limited, particularly since hunting is prohibited in the BINP” (lines 382-383), and reasons for eating meat are “extreme hunger due to poverty and potential economic loss” (lines 399-400). This means that individuals may well be very aware that they are at risk of disease transmission if eating meat from sick animals, but do so anyway due to the value of meat or lack of available food. In such a case, it is hard to say how further education or awareness raising would resolve this situation. I would have thought that results such as this point to the intractable and systemic nature of problems related to the specific context of the study, but which may have echoes in other settings – and therefore point to the need for a more contextualised policy approach which takes systemic factors into account, or to include field actors to a greater extent in the construction of policy.

I note that reviewer 2 also made similar comments on the discussion – I would encourage the authors to think more deeply about how their findings could be used to inform the development and implementation of locally-relevant diseases management/mitigation policies, the importance of engaging with field actors, and the feasibility of implementing strategies for change in communities which face intractable problems such as poverty and poor access to health services. The authors do not have to come up with the solutions to these very challenging problems! But they do need to openly acknowledge the impact such factors have on disease risk mitigation and provide some thought on their impact for making use of their results. This paper has a lot of useful information and it would be a shame to suggest the only conclusion it has to offer is to improve education.

**Response:** The authors really appreciate these sentiments and agree. We’ve updated the discussion and conclusions accordingly.

We believe these revisions have significantly strengthened the manuscript and we hope it is now suitable for publication in *PLOS Neglected Tropical Diseases*. Thank you again for your guidance and for considering our revised manuscript.

Sincerely,

**Nahabwe Haven**

[**hahotice@googlemail.com**](mailto:hahotice@googlemail.com)
Corresponding Author
On behalf of all co-authors
